# DNA methylation changes at infertility genes in newborn twins conceived by *in vitro* fertilisation

**DOI:** 10.1186/s13073-017-0413-5

**Published:** 2017-03-24

**Authors:** Juan E. Castillo-Fernandez, Yuk Jing Loke, Sebastian Bass-Stringer, Fei Gao, Yudong Xia, Honglong Wu, Hanlin Lu, Yuan Liu, Jun Wang, Tim D. Spector, Richard Saffery, Jeffrey M. Craig, Jordana T. Bell

**Affiliations:** 1 Department of Twin Research and Genetic Epidemiology, King’s College London, SE1 7EH London, UK; 2 Early Life Epigenetics, Murdoch Childrens Research Institute, Royal Children’s Hospital, Parkville, VIC Australia; 3 BGI-Shenzhen, Shenzhen, China; 4 King Abdulaziz University, Jeddah, 22254 Saudi Arabia; 5 Department of Biology, University of Copenhagen, Copenhagen, DK-2200 Denmark; 6 The Novo Nordisk Foundation Center for Basic Metabolic Research, University of Copenhagen, Copenhagen, DK-2200 Denmark; 7 Department of Paediatrics, University of Melbourne, Parkville, VIC Australia; 8 Cancer, Disease and Developmental Epigenetics, Murdoch Childrens Research Institute, Royal Children’s Hospital, Parkville, VIC Australia

**Keywords:** *In vitro* fertilisation (IVF), Epigenomics, DNA methylation, MeDIP-seq

## Abstract

**Background:**

The association of *in vitro* fertilisation (IVF) and DNA methylation has been studied predominantly at regulatory regions of imprinted genes and at just thousands of the ~28 million CpG sites in the human genome.

**Methods:**

We investigated the links between IVF and DNA methylation patterns in whole cord blood cells (*n* = 98) and cord blood mononuclear cells (*n* = 82) from newborn twins using genome-wide methylated DNA immunoprecipitation coupled with deep sequencing.

**Results:**

At a false discovery rate (FDR) of 5%, we identified one significant whole blood DNA methylation change linked to conception via IVF, which was located ~3 kb upstream of *TNP1*, a gene previously linked to male infertility. The 46 most strongly associated signals (FDR of 25%) included a second region in a gene also previously linked to infertility, *C9orf3*, suggesting that our findings may in part capture the effect of parental subfertility. Using twin modelling, we observed that individual-specific environmental factors appear to be the main overall contributors of methylation variability at the FDR 25% IVF-associated differentially methylated regions, although evidence for methylation heritability was also obtained at several of these regions. We replicated previous findings of differential methylation associated with IVF at the *H19/IGF2* region in cord blood mononuclear cells, and we validated the signal at *C9orf3* in monozygotic twins. We also explored the impact of intracytoplasmic sperm injection on the FDR 25% signals for potential effects specific to male or female infertility factors.

**Conclusions:**

To our knowledge, this is the most comprehensive study of DNA methylation profiles at birth and IVF conception to date, and our results show evidence for epigenetic modifications that may in part reflect parental subfertility.

**Electronic supplementary material:**

The online version of this article (doi:10.1186/s13073-017-0413-5) contains supplementary material, which is available to authorized users.

## Background

As the frequency of *in vitro* fertilisation (IVF) treatment increases worldwide, much research effort has focused on exploring both short- and long-term health outcomes associated with conception via IVF, with contradictory results. A number of studies have observed associations with adverse perinatal and obstetric outcomes, including low birth weight, preterm birth, perinatal mortality, congenital malformations, placental complications, and increased frequency of imprinting disorders such as Angelman syndrome and Beckwith-Wiedemann syndrome [1–4]. On the other hand, parallel efforts have reported that these associations are not attributed to IVF treatment itself, but rather to multiple pregnancy or parental subfertility, both common factors in IVF births [5, 6]. Further research is required to identify potential factors associated with conception via IVF, including not only health outcomes but also biological consequences such as epigenetic modifications.

Given that birth weight and imprinting disorders are controlled at least in part by epigenetic factors [7, 8], IVF may have an influence on epigenetic profiles, potentially resulting in changes that persist well after birth and over the life course. Epigenetic mechanisms are considered possible mediators of the developmental origins of health and disease [9]; therefore, an assessment of the influence of IVF on DNA methylation profiles may give some insights into mechanisms underlying potential related health outcomes. Establishment of DNA methylation profiles in the germ line and embryo takes place early in development [10]. Theoretically, this epigenetic reprogramming could therefore be influenced by IVF-related interventions that occur very early, prior to blastocyst implantation. Indeed, induction of ovulation, embryo culturing, and cryopreservation, among others, have all been linked to specific alterations in DNA methylation in mice, although results are somewhat inconsistent [11–13].

Most studies in humans comparing naturally and IVF-conceived newborns have interrogated DNA methylation alterations targeting almost exclusively imprinted differentially methylated regions (DMRs). These studies have reported increased epigenetic variability at the *KvDMR1*, *PEG1*, and *H19* DMRs in umbilical cord blood [14], hypomethylation of the *H19* and *MEST* DMRs in placenta [15], and hypomethylation of the *H19* DMR in buccal epithelium [16] in individuals conceived by IVF. High-throughput approaches using bead array technology have also interrogated DNA methylation in IVF in a genome-wide manner. Katari et al. [17] reported differential methylation at 78 genes in cord blood and 40 in placenta with at least two differentially methylated CpG sites (*P* ≤ 0.08) when looking across the promoters of 736 genes (GoldenGate Array, Illumina) in ten cases and 13 controls. A more extensive study using the promoter-enriched Illumina Infinium HumanMethylation27 bead array in cord blood samples from ten IVF cases and eight controls reported a total of 24 genes with at least two differentially methylated CpG sites (*P* < 0.05) [18]. More recently, a study used the genome-wide Illumina Infinium HumanMethylation450 bead array in samples from 38 IVF-conceived newborns followed by fresh embryo transfer, 38 IVF-conceived followed by cryopreserved embryo transfer, 18 born to subfertile parents after conception by intrauterine insemination, and 43 controls born to fertile parents [19]. This platform interrogates CpG sites across the whole genome, although with a limited coverage since it targets gene-centric annotations [20]. The authors identified differential methylation at multiple sites, including metastable epialleles.

Here, we interrogated evidence for differential methylation between IVF and non-IVF newborn twins in a more comprehensive manner by conducting epigenome-wide association scans (EWAS) [21] using methylated DNA immunoprecipitation followed by deep sequencing (MeDIP-seq) [22] genome-wide in samples from cord blood, and its mononuclear fraction, collected at birth from IVF and non-IVF twins. The use of twins in this study allowed the partition of the observed variance in DNA methylation into genetic and environmental factors. The approach also avoids potential spurious associations due to an imbalanced number of multiple and single pregnancies between conception method groups.

## Methods

### Subjects and sample collection

The study included 47 IVF and 60 non-IVF newborn twins (from 54 twin pairs) from the Peri/postnatal Epigenetic Twins Study (PETS), Melbourne, Australia. Recruitment and full study procedure have been described previously [23, 24]. Cord blood was collected at birth and used to process mononuclear cells by Ficoll gradient centrifugation as described previously [25]. Whole blood cells (WBCs) from cord blood were available for a total of 98 twins (40 IVF and 58 non-IVF) and cord blood mononuclear cells (CBMCs) for a total of 82 twins (35 IVF and 47 non-IVF). Maternal age and method of conception were determined via questionnaire at recruitment (18–20 weeks gestation). Twins of mothers who said yes to IVF or intracytoplasmic sperm injection (ICSI) treatment were classified as IVF regardless of the use of ovulation induction medication or other fertility treatments. Maternal smoking status was collected via questionnaire on recruitment and at 24 and 36 weeks of pregnancy. Birth weight was collected during the immediate neonatal period. Zygosity and chorionicity were determined by physical examination of the inter-placental membranes at birth, and by genetic test when required, as described previously [23, 24]. Pregnancy complications were recorded and are shown in Additional file 1: Table S1.

### DNA methylation profiling

MeDIP-seq was performed at BGI-Shenzhen, Shenzhen, China. Extracted DNA was fragmented using a Covaris sonication system and sequencing libraries were prepared from 5 μg fragmented genomic DNA. End repair, <A > base addition and adaptor ligation steps were performed using Illumina’s Single-End DNA Sample Prep kit. Adaptor-ligated DNA was immunoprecipitated by anti-5mC using a commercial antibody (Diagenode) and MeDIP products were validated by quantitative PCR. MeDIP DNA was purified with ZYMO DNA Clean & Concentrator-5 columns and amplified using adaptor-mediated PCR. DNA fragments between 200 and 500 bp in size were gel-excised, and the amplification quality and quantity were evaluated by Agilent BioAnalyzer analysis. The libraries were subjected to highly parallel 50-bp single-end sequencing on the Illumina GAII platform. All sequencing data passed initial quality checks for base composition (no exclusions) using FASTQC v0.10.0. For each individual, ~30 million reads were generated and mapped onto hg19 using BWA. After removing duplicates, we filtered data using quality score Q10. We quantified methylation levels using MEDIPS [26], producing the mean relative methylation score (RPM) in 500-bp bins (overlap of 250 bp) across the genome. Altogether, there were 11,524,145 windows and these were used for the analyses. Bins with RPM values of zero in more than 50% of the samples were excluded, resulting in 9,592,803 (WBC) and 9,285,089 (CBMC) bins used in downstream analyses.

### Epigenome-wide IVF-DMR analyses

Normalised (N(0,1)) methylation scores in each genomic bin were regressed using a linear mixed-effects model to account for twin structure (lme4 package [27] in R [28]). Tissue type, birth weight, sex, maternal smoking, 260/280 ratio, DNA concentration, and the loadings of the first five principal components were used as covariates and included as fixed effects in the model. Family and zygosity were included as random effects in the linear mixed model. The principal components were included to account for unknown sources of variation, such as cell heterogeneity. Correction for multiple testing was performed by a Benjamini-Hochberg false discovery rate (FDR) calculation.

### Variance decomposition of WBC IVF-DMRs

The contribution of additive genetic (A), common environmental (C), and unique environmental (E) factors to DNA methylation was estimated using the ACE model based on the classic twin design [29]. The model was fitted using the OpenMX statistical package [30]. RPM values without adjustment for covariates were used to estimate the ACE proportions.

### Statistical analysis

Pairwise correlations and principal components analysis were performed using RPM values across all bins with values > 0 in at least 50% of the samples. Hierarchical clustering was performed using Euclidean distance as a measure of dissimilarity and average linkage clustering.

### Validation analysis

Genomic DNA (500 ng) was bisulphite converted using the MethylEasy Exceed Rapid Bisulphite Modification Kit (Human Genetic Signatures, North Ryde, NSW, Australia). Primers to target the regions in *TNP1* and *C9orf3* were designed using the EpiDesigner tool (Sequenom Inc., Herston, QLD, Australia). The *H19 CTCF6* region was the same used in a previous study [25]. Primers, genomic coordinates, and PCR conditions are shown in Additional file 1: Table S2. Methylation levels were determined by EpiTYPER on the MassARRAY System (Sequenom Inc., Herston, QLD, Australia). Statistical analysis considered the average of two to three technical replicates and were performed using data on single CpG sites.

## Results

### Genome-wide methylation profiles in twins

**Fig. 1:**
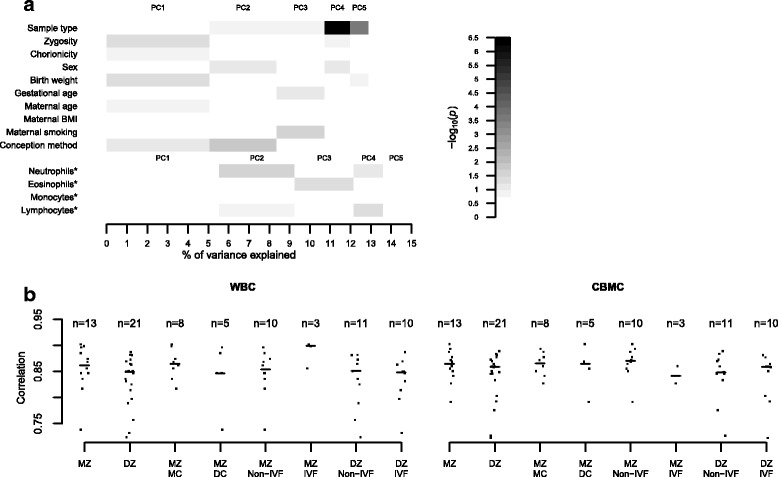
Global methylation patterns. **a** Biological factors associated with principal components of variation of methylation profiles. Variables marked with an *asterisk* were only available in a subset of the sample (*n* = 54). **b** Within-pair methylation correlation in WBCs and CBMCs. *BMI* body mass index, *DC* dichorionic, *DZ* dizygotic, *MC* monochorionic, *MZ* monozygotic, *PC* principal component

We profiled DNA methylation levels from a total of 107 newborn twins (47 conceived via IVF and 60 conceived in vivo) in WBCs and CBMCs. Details of any fertility treatment used and demographic characteristics that represent potential confounders of DNA methylation levels at birth, such as sex, birth weight, maternal age, and maternal smoking status, are shown in Table 1. We first explored the genome-wide patterns of DNA methylation variability in the dataset. Principal component analysis was used to identify factors that were significantly associated with genome-wide variability in DNA methylation profiles. The first five principal components in the dataset, which explained ~13% of the total variance in DNA methylation, were at least nominally associated (*P* < 0.05) with sample type (WBCs versus CMBCs), birth weight, maternal smoking, and conception method (Fig. 1a).

**Table 1:** Breakdown of samples used for the identification of IVF-DMRs and potential covariates

Group	Total number of twins (number of complete sets)	Zygosity and chorionicity^a^	Sex^b^	Birth weight (kg): mean(sd)	Maternal age (years): mean (sd)	Maternal smoking (percentage smokers)	Ovarian stimulation	ICSI	GIFT	Frozen embryo
WBCs										
IVF	40 (20)	10 MZ MC 30 DZ DC	18 (F) 22 (M)	2.57 (4.77)	36 (4)	20%	6 (No) 34 (Yes)	22 (No) 18 (Yes)	-	28 (No) 12 (Yes)
Non-IVF	58 (29)	14 MZ MC 12 MZ DC 32 DZ DC	34 (F) 24 (M)	2.58 (3.99)	32 (5)	28%	56 (No) 2 (Yes)	-	56 (No) 2 (Yes)	-
CBMCs										
IVF	35 (16)	9 MZ MC 1 MZ DC 25 DZ DC	16 (F) 19 (M)	2.50 (4.42)	35 (5)	23%	1 (No) 34 (Yes)	14 (No) 21 (Yes)	-	24 (No) 11 (Yes)
Non-IVF	47 (22)	12 MZ MC 10 MZ DC 25 DZ DC	30 (F) 17 (M)	2.60 (3.48)	32 (4)	28%	45 (No) 2 (Yes)	-	45 (No) 2 (Yes)	-

^a^ *MZ* monozygotic, *DZ* dizygotic, *MC* monochorionic, *DC* dichorionic

^b^ *F* female, *M* male

*GIFT* gamete intra-fallopian transfer, *ICSI* intracytoplasmic sperm injection, *sd* standard deviation

We next estimated the within twin-pair correlation patterns in methylation profiles of twin pairs available in both datasets using Pearson’s correlation. In concordance with previous studies [7], we observed higher median correlation within monozygotic (MZ) twin pairs compared to dizygotic (DZ) twin pairs (Fig. 1b). Previous studies have shown that twin chorionicity can have an effect on within-pair DNA methylation differences, but not with consistent direction of effect across tissues [7, 25, 31]. In our study, we did not observe significant chorionicity-related methylation differences (Fig. 1b), but the number of MZ twins within chorionicity categories was relatively low (*n* = 8 monochorionic and *n* = 5 dichorionic pairs). Interestingly, the method of conception showed methylation profile differences within MZ twin pairs. MZ IVF twins had higher median correlation compared to MZ non-IVF twins in WBCs, but the opposite trend was observed in CBMCs, and in both cases the MZ IVF sample was very small (*n* = 3).

### IVF-DMRs in CBMCs and WBCs

**Fig. 2:**
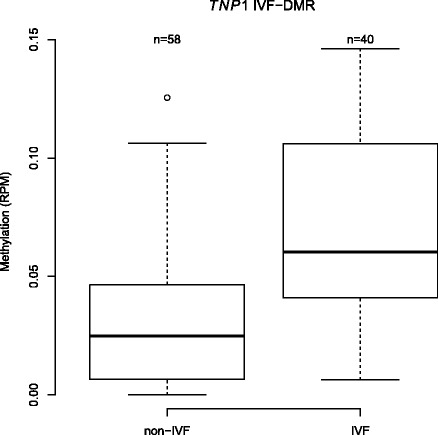
*TNP1* IVF-DMR. Methylation values (RPM) at the top IVF-DRM identified ~3 kb upstream of *TNP1* in WBCs

In order to identify tissue-independent and tissue-specific IVF-associated DMRs, we compared DNA methylation profiles in WBCs and CBMCs in relation to method of conception adjusting for birth weight, sex, maternal smoking, and the first five principal components, which partly capture cell heterogeneity. Epigenome-wide analyses of DNA methylation in relation to method of conception did not identify genome-wide significant signals in the CBMCs subset or in the combined CBMC and WBC datasets, after correction for multiple testing. In WBCs alone, one significant DMR was observed at a FDR of 5% (Fig. 2). This was located ~3 kb upstream of *TNP1* (chr2:217,726,751–217,727,250), which encodes a transition nuclear protein that replaces histones and is subsequently replaced by protamines during spermiogenesis. A deletion in the promoter region of this gene, which reduces its expression, has been reported in infertile men [32]. Methylation upstream of *TNP1* might have an impact on its expression. In mice, methylation changes during spermatogenesis have been observed at *TNP1*, which suggests a role of methylation in the regulation of this gene [33]. To explore the biological characteristics of the top-ranked results in the IVF epigenome-wide analyses we selected a more liberal threshold of FDR 25%, at which 46 IVF-DMRs were identified (Table 2). Interestingly, the third-ranked DMR genome-wide (Additional file 1: Figure S1) was located in the first intron of *C9orf3* (chr9:97,504,001–97,504,500), which has been associated with polycystic ovary syndrome in women [34] and development of erectile dysfunction after radiotherapy for prostate cancer in men [35]. Another signal within this list was located in intron 1 of *STOX2* (chr4:184,814,001–184,814,500), whose reduced expression has been implicated in pre-eclampsia [36]. Since adverse perinatal outcomes may be associated with maternal age, we further adjusted for this covariate and observed that the 46 FDR 25% WBC IVF-DMRs remained significant (Table 2).

**Table 2:** FDR 25% WBC IVF-DMRs

Chromosome	Start	End	*P*	FDR adjusted *P*	*P* (adjusted for maternal age)	Gene name^a^	Gene start^a^	Gene end^a^
Chr2	217726751	217727250	2.30E-09	0.0221	6.40E-10	*AC007557.1*	217735495	217736362
						*TNP1*	217724181	217724787
Chr5	178761751	178762250	5.43E-08	0.1244	1.76E-05	*ADAMTS2*	178537852	178772431
Chr9	97504001	97504500	5.83E-08	0.1244	5.14E-07	*C9orf3*	97488983	97849441
Chr5	9275751	9276250	5.86E-08	0.1244	1.67E-06	*SEMA5A*	9035138	9546187
Chr4	184814001	184814500	7.95E-08	0.1244	4.96E-07	*STOX2*	184774584	184944679
Chr5	142488501	142489000	8.73E-08	0.1244	5.76E-07	*ARHGAP26*	142149949	142608576
Chr9	118148751	118149250	9.20E-08	0.1244	4.61E-06	*DEC1*	117904097	118164923
Chr9	118149001	118149500	1.04E-07	0.1244	3.47E-06	*DEC1*	117904097	118164923
Chr11	82654251	82654750	1.30E-07	0.1250	5.26E-08	*C11orf82*	82611017	82669319
						*PRCP*	82534544	82681626
						*RAB30*	82684175	82782965
Chr19	6165251	6165750	1.40E-07	0.1250	1.00E-06	*RFX2*	5993175	6199583
						*ACSBG2*	6135258	6193112
						*MLLT1*	6212966	6279959
Chr1	85522251	85522750	1.43E-07	0.1250	2.56E-07	*WDR63*	85464830	85598821
						*MCOLN3*	85483765	85514182
Chr17	42569001	42569500	1.64E-07	0.1274	1.90E-05	*GPATCH8*	42472652	42580798
Chr4	141606501	141607000	2.03E-07	0.1274	1.59E-05	*TBC1D9*	141541919	141677274
Chr5	137736001	137736500	2.06E-07	0.1274	1.13E-06	*REEP2*	137774706	137782658
						*KDM3B*	137688285	137772717
Chr5	150614501	150615000	2.14E-07	0.1274	2.23E-07	*SLC36A3*	150656323	150683327
						*GM2A*	150591711	150650001
						*CCDC69*	150560613	150603706
Chr17	36918251	36918750	2.32E-07	0.1274	2.02E-07	*MLLT6*	36861795	36886056
						*CISD3*	36886488	36891297
						*CWC25*	36956687	36981734
						*PIP4K2B*	36921942	36956379
						*PCGF2*	36890150	36906070
						*CTB-58E17.5*	36905613	36906969
						*PSMB3*	36908989	36920484
						*AC006449.1*	36884086	36884451
Chr6	126138251	126138750	2.36E-07	0.1274	2.12E-06	*NCOA7*	126102307	126252266
Chr7	144431251	144431750	2.70E-07	0.1274	5.29E-09	*TPK1*	144149034	144533488
Chr12	70937251	70937750	2.76E-07	0.1274	1.83E-06	*PTPRB*	70910630	71031220
Chr4	141606251	141606750	2.80E-07	0.1274	1.50E-05	*TBC1D9*	141541919	141677274
Chr13	90019001	90019500	2.84E-07	0.1274	5.94E-07	*-*	-	-
Chr11	74179001	74179500	2.92E-07	0.1274	1.61E-09	*LIPT2*	74202757	74204778
						*POLD3*	74204896	74380162
						*KCNE3*	74165886	74178774
Chr12	99153001	99153500	3.93E-07	0.1637	2.44E-07	*ANKS1B*	99120235	100378432
						*APAF1*	99038919	99129204
Chr2	223336751	223337250	4.47E-07	0.1788	2.00E-06	*SGPP2*	223289236	223425667
Chr8	120972001	120972500	4.98E-07	0.1869	3.26E-08	*DEPTOR*	120885957	121063152
Chr17	38047001	38047500	5.25E-07	0.1869	7.83E-06	*GSDMB*	38060848	38076107
						*ZPBP2*	38024417	38034149
						*IKZF3*	37921198	38020441
						*ORMDL3*	38077294	38083854
Chr4	64626751	64627250	5.45E-07	0.1869	2.67E-06	*-*	-	-
Chr16	87256751	87257250	5.51E-07	0.1869	2.12E-08	*C16orf95*	87117168	87351022
Chr19	10656751	10657250	5.77E-07	0.1869	1.42E-06	*CDKN2D*	10677138	10679735
						*ATG4D*	10654571	10664094
						*KEAP1*	10596796	10614417
						*AP1M2*	10683347	10697991
						*KRI1*	10663761	10676713
						*S1PR5*	10623623	10628607
Chr7	2487251	2487750	5.85E-07	0.1869	2.03E-05	*CHST12*	2443223	2474242
Chr11	74178751	74179250	6.82E-07	0.2059	1.31E-07	*KCNE3*	74165886	74178774
						*LIPT2*	74202757	74204778
						*POLD3*	74204896	74380162
Chr10	119176501	119177000	6.87E-07	0.2059	1.11E-06	*PDZD8*	119040000	119134978
Chr22	34755251	34755750	7.32E-07	0.2094	1.76E-05	*-*	-	-
Chr6	161664751	161665250	7.42E-07	0.2094	2.93E-06	*AGPAT4*	161551011	161695093
Chr16	17161751	17162250	7.80E-07	0.2137	2.41E-06	*XYLT1*	17195626	17564738
Chr18	23695001	23695500	8.39E-07	0.2235	1.97E-06	*PSMA8*	23713816	23773319
						*SS18*	23596578	23671181
Chr9	26364751	26365250	8.90E-07	0.2267	1.02E-06	*-*	-	-
Chr1	25227001	25227500	9.05E-07	0.2267	1.61E-05	*RUNX3*	25226002	25291612
Chr13	68877251	68877750	9.43E-07	0.2267	2.19E-05	*-*	-	-
Chr9	89126501	89127000	9.59E-07	0.2267	7.00E-06	*-*	-	-
Chr13	35317501	35318000	9.72E-07	0.2267	2.51E-05	*-*	-	-
Chr21	19575001	19575500	9.92E-07	0.2267	2.31E-06	*CHODL*	19273580	19639690
Chr2	169470001	169470500	1.06E-06	0.2307	4.35E-07	*CERS6*	169312372	169631644
Chr12	4310251	4310750	1.06E-06	0.2307	8.13E-05	*-*	-	-
Chr6	157136501	157137000	1.15E-06	0.2448	1.61E-06	*ARID1B*	157099063	157531913
Chr14	104067251	104067750	1.17E-06	0.2448	8.21E-06	*APOPT1*	104029299	104073860
						*BAG5*	104022881	104029168
						*KLC1*	104028233	104167888
						*RP11-73 M18.2*	104029299	104152261

^a^From GENCODE v19

The non-IVF group included a small number (*n* = 4) of newborns conceived with other types of fertility treatments not equivalent to IVF, such as gamete intra-fallopian transfer (GIFT) and ovarian stimulation. We re-analysed the 46 FDR 25% WBC IVF-DMRs excluding GIFT (*n* = 2) and non-IVF ovarian stimulation (*n* = 2) controls and observed that conclusions remained unchanged (Additional file 1: Table S3).

**Fig. 3:**
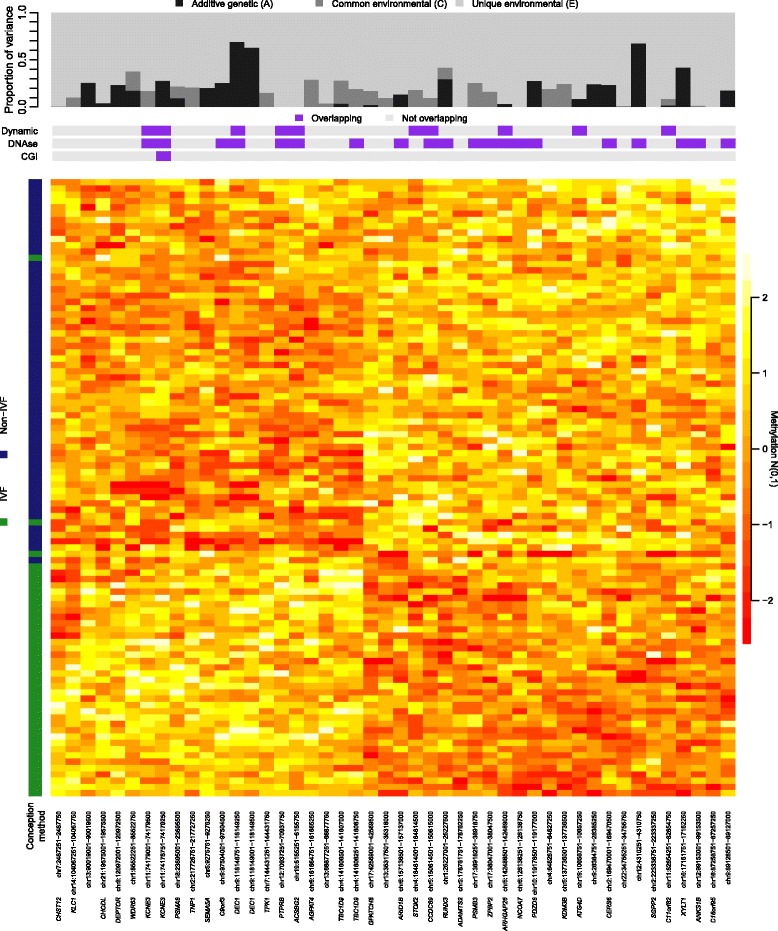
WBC IVF-DMRs. Heatmap rows correspond to the 98 WBC samples while columns correspond to the 46 FDR 25% WBC IVF-DMRs. The *vertical colour bar* indicates method of conception (IVF, *green*; non-IVF, *blue*). *Top panel* shows the fraction of variance explained by additive genetic (A), shared environmental (C), and unique environmental (E) factors. *Horizontal colour bars* indicate overlap (*violet*) or absence (*gray*) of dynamic CpG sites, DNase I hypersensitivity sites, or CpG islands with the DMR

Hierarchical clustering using DNA methylation levels at these 46 FDR 25% DMRs alone grouped twins by method of conception, assigning 38 out of 40 IVF twins and 57 out of 58 non-IVF twins to the correct group (Fig. 3). We also explored these signals with respect to functional annotations. A total of ten FDR 25% WBC IVF-DMRs overlapped CpG sites previously shown to be dynamic during development [37], 20 overlapped DNase I hypersensitivity sites (wgEncodeRegDnaseClusteredV3) [38], one overlapped a CpG island (cpgIslandEx) [39], and none overlapped with candidate metastable epialleles [40] (Fig. 3).

Cell type-specific DNA methylation can impact the profiles observed in a population of cells, such as in a whole blood sample, and we therefore accounted for blood cell type heterogeneity using a twofold approach. First, we performed principal component analysis on the methylation levels of the entire set of WBC samples, and our main EWAS analyses above are corrected for the first five principal components, which likely capture variation attributed to technical and biological factors, potentially including cell heterogeneity. To assess whether the first five principal components capture cell heterogeneity, blood cell subtype counts were obtained through automatic differential counting for a subset of the WBC samples (*n* = 54 twins, 22 IVF, and 32 non-IVF) and these were compared against the distributions of the first five principal components. The proportion of neutrophils, eosinophils, and lymphocytes were associated (*P* < 0.05) with the loadings of the second, third, and fourth principal components, respectively (Fig. 1a). Therefore, since the EWAS model used in this study took into account the loadings of the first five principal components, these analyses already take into account the influence of cell heterogeneity to a certain extent.

Second, we re-analysed the 46 FDR 25% WBC IVF-DMRs in the subset of 54 WBC samples with available cell counts, adjusting for the proportion of neutrophils, eosinophils, monocytes, and lymphocytes. We also performed analyses adjusting for the loadings of the first five principal components within this dataset alone. Most results were concordant when comparing across all models (Additional file 1: Table S4) and only five out the 46 FDR 25% WBC IVF-DMRs were not significant (*P* > 0.05) after adjusting for cell proportions (chr8:120,972,001–120,972,500, chr7:2,487,251–2,487,750, chr18:23,695,001–23,695,500, chr12:4,310,251–4,310,750, and chr14:104,067,251–104,067,750).

### Variance decomposition of WBC IVF-DMRs

Given that epigenetic changes were potentially affecting infertility genes, we wanted to investigate if the findings may capture a genetic signature affecting DNA methylation that could be transmitted to offspring. We applied twin variance decomposition analyses to partition the total epigenetic variance into additive genetic (A) and common (C) and unique (E) environmental components (ACE) [29]. The ACE model was used to determine the contribution of genetics, shared intrauterine environment due to shared maternal influences, and non-shared (twin-specific) or stochastic factors to epigenetic variation. The mean contribution of additive genetic effects (narrow-sense heritability) to DNA methylation across the genome in different tissues from newborns has been previously estimated to be between 0.05 and 0.12 [7]. Here we estimated the average genome-wide narrow-sense heritability for DNA methylation in WBCs at 0.06. At the 46 FDR 25% WBC IVF-DMRs, the major contributors to DNA methylation variation were non-shared or stochastic events (Fig. 3). However, several FDR 25% IVF-DMRs had evidence for heritability (A > 0.4), suggestive of genetic effects underlying specific IVF-associated DNA methylation changes. These included an intronic region in *DEC1* (chr9:118,148,751–118,149,500), a region 33 kb away from *XYLT1* (chr16:17,161,751–17,162,250), and an intergenic region in chromosome 12 (chr12:4,310,251–4,310,750). When looking at the two DMRs associated with infertility genes, DNA methylation variation showed no evidence for genetic effects (A = 0) near *TNP1*, while heritability at the DMR in *C9orf3* was estimated at 0.25.

### Effects of IVF on imprinting

Previous studies have explored DNA methylation patterns in IVF births specifically at imprinting control regions (ICRs). We therefore assessed whether there was an enrichment of differential methylation effects at 34 known ICRs [41] in our genome-wide results, but no enrichment was observed (*P* > 0.05). However, when we explored individual signals at candidate IVF-DMRs we were able to replicate one previously reported ICR IVF-associated DMR. Concordantly with previous IVF methylation studies in placental tissue [15] and buccal epithelium [16], we observed hypomethylation in IVF twins at the sixth CTCF binding site within the *H19*/*IGF2* (*H19 CTCF6*) DMR (Additional file 1: Figure S2). This association was observed in CBMCs (*P* = 0.01), but not in WBCs.

### Effects of ICSI

ICSI is a technique in IVF used to treat couples with male-factor infertility [42]. In contrast to conventional IVF where fertilisation occurs by placing spermatozoa near an egg, ICSI consists of the direct injection of a selected single sperm cell into the egg. This manipulation may introduce additional risk factors [43]. To assess the effect of ICSI on the 46 FDR 25% WBC IVF-DMRs we adjusted for the use of this technology and also compared the ICSI and the conventional IVF groups separately against the non-IVF group. After adjustment for ICSI, the association weakened at several FDR 25% IVF-DMRs (Table 3), suggesting that ICSI or paternal infertility might have a role in these methylation changes. One FDR 25% IVF-DMR signal (chr1:85,522,251–85,522,750) appeared stronger after adjustment, suggesting either a female infertility effect or that ICSI prevents or corrects a methylation change that occurs in conventional IVF. This DMR was located upstream of *WDR63*, a gene mainly expressed in testis, fallopian tube, and adrenal gland [44].

**Table 3:** Effect of ICSI on the FDR 25% WBC IVF-DMRs

			IVF (*n* = 40) versus non-IVF (*n* = 58)			IVF (*n* = 34) versus non-IVF (*n* = 58) adjusted for ICSI			Conventional IVF (*n* = 16) versus non-IVF (*n* = 58)			ICSI (*n* = 18) versus non-IVF (*n* = 58)		
Chromosome	Start	End	Estimate	SE	*P*	Estimate	SE	*P*	Estimate	SE	*P*	Estimate	SE	*P*
Chr2	217726751	217727250	1.18	0.19	2.30E-09	1.45	0.26	7.71E-08	−1.42	0.26	7.44E-08	−1.16	0.27	1.29E-05
Chr5	178761751	178762250	−1.08	0.2	5.43E-08	−1.09	0.29	8.23E-05	0.87	0.30	1.89E-03	1.11	0.27	2.52E-05
Chr9	97504001	97504500	1.07	0.2	5.83E-08	0.77	0.27	2.77E-03	−0.88	0.31	2.67E-03	−1.30	0.25	1.95E-07
Chr5	9275751	9276250	1.09	0.19	5.86E-08	1.17	0.27	1.04E-05	−1.13	0.29	6.50E-05	−1.13	0.27	2.75E-05
Chr4	184814001	184814500	−0.96	0.18	7.95E-08	−0.97	0.24	3.94E-05	0.94	0.26	1.37E-04	1.15	0.23	8.35E-07
Chr5	142488501	142489000	−1.11	0.2	8.73E-08	−1.1	0.28	7.60E-05	1.25	0.31	5.16E-05	1.08	0.28	7.10E-05
Chr9	118148751	118149250	1.09	0.2	9.20E-08	1.35	0.28	1.28E-06	−1.30	0.29	8.90E-06	−1.02	0.28	1.68E-04
Chr9	118149001	118149500	1.08	0.2	1.04E-07	1.31	0.28	2.22E-06	−1.23	0.29	2.00E-05	−1.05	0.29	1.69E-04
Chr11	82654251	82654750	−1.05	0.19	1.30E-07	−0.97	0.29	4.68E-04	0.95	0.31	1.53E-03	0.88	0.27	5.94E-04
Chr19	6165251	6165750	0.94	0.18	1.40E-07	0.89	0.25	1.90E-04	−0.88	0.28	9.35E-04	−1.02	0.23	7.36E-06
Chr1	85522251	85522750	0.99	0.18	1.43E-07	1.54	0.25	4.48E-09	−1.53	0.25	7.41E-09	−0.80	0.28	2.32E-03
Chr17	42569001	42569500	−1.07	0.19	1.64E-07	−1.25	0.27	2.21E-06	1.30	0.28	3.81E-06	1.21	0.26	2.44E-06
Chr4	141606501	141607000	1.09	0.19	2.03E-07	1.26	0.26	3.56E-06	−1.33	0.30	1.05E-05	−1.13	0.26	1.83E-05
Chr5	137736001	137736500	−1.03	0.19	2.06E-07	−1.11	0.28	4.12E-05	1.13	0.30	1.39E-04	1.10	0.27	3.78E-05
Chr5	150614501	150615000	−1.08	0.21	2.14E-07	−1.2	0.29	2.80E-05	1.24	0.31	3.18E-05	1.07	0.29	1.21E-04
Chr17	36918251	36918750	−1.1	0.21	2.32E-07	−0.96	0.29	6.01E-04	1.05	0.31	4.88E-04	1.03	0.28	1.83E-04
Chr6	126138251	126138750	−0.99	0.19	2.36E-07	−0.75	0.27	3.29E-03	0.79	0.30	4.92E-03	1.22	0.25	8.94E-07
Chr7	144431251	144431750	1.02	0.19	2.70E-07	0.84	0.28	1.30E-03	−0.85	0.28	1.48E-03	−1.17	0.27	1.77E-05
Chr12	70937251	70937750	0.88	0.17	2.76E-07	0.72	0.23	1.12E-03	−0.87	0.26	5.26E-04	−0.94	0.20	3.10E-06
Chr4	141606251	141606750	1.01	0.19	2.80E-07	1.16	0.26	8.05E-06	−1.31	0.29	6.93E-06	−1.04	0.25	2.14E-05
Chr13	90019001	90019500	1.06	0.2	2.84E-07	1.25	0.29	8.88E-06	−1.36	0.30	5.09E-06	−0.84	0.29	2.20E-03
Chr11	74179001	74179500	1.07	0.2	2.92E-07	1.3	0.29	7.25E-06	−1.37	0.29	3.15E-06	−1.06	0.29	1.76E-04
Chr12	99153001	99153500	−1.01	0.2	3.93E-07	−1.02	0.29	2.02E-04	1.02	0.31	4.59E-04	1.24	0.27	3.77E-06
Chr2	223336751	223337250	−1.01	0.2	4.47E-07	−1.41	0.28	4.00E-07	1.50	0.29	1.69E-07	0.77	0.29	4.65E-03
Chr8	120972001	120972500	0.97	0.19	4.98E-07	0.83	0.27	1.22E-03	−0.87	0.29	1.43E-03	−0.96	0.26	1.81E-04
Chr17	38047001	38047500	−1.04	0.2	5.25E-07	−1.3	0.28	4.51E-06	1.31	0.27	1.21E-06	0.74	0.28	5.39E-03
Chr4	64626751	64627250	−0.99	0.19	5.45E-07	−0.87	0.28	9.92E-04	0.86	0.29	1.61E-03	0.98	0.26	1.36E-04
Chr16	87256751	87257250	−0.83	0.16	5.51E-07	−0.97	0.23	1.75E-05	0.94	0.25	9.13E-05	0.82	0.24	3.37E-04
Chr19	10656751	10657250	−1.03	0.2	5.77E-07	−1.09	0.28	6.47E-05	1.17	0.29	3.80E-05	1.13	0.27	2.18E-05
Chr7	2487251	2487750	0.8	0.16	5.85E-07	0.73	0.23	1.06E-03	−0.81	0.24	5.61E-04	−0.94	0.23	3.49E-05
Chr11	74178751	74179250	0.99	0.2	6.82E-07	1.19	0.28	2.15E-05	−1.15	0.28	2.45E-05	−0.82	0.28	2.37E-03
Chr10	119176501	119177000	−1.04	0.2	6.87E-07	−1.03	0.29	3.41E-04	1.16	0.31	1.69E-04	1.14	0.28	4.83E-05
Chr22	34755251	34755750	−0.98	0.2	7.32E-07	−0.8	0.28	2.35E-03	0.97	0.31	1.21E-03	1.15	0.25	3.95E-06
Chr6	161664751	161665250	0.98	0.19	7.42E-07	0.9	0.26	3.89E-04	−0.83	0.29	2.48E-03	−0.81	0.25	7.71E-04
Chr16	17161751	17162250	−1.01	0.2	7.80E-07	−0.99	0.28	2.19E-04	1.17	0.30	4.48E-05	1.08	0.28	1.16E-04
Chr18	23695001	23695500	1.04	0.21	8.39E-07	0.95	0.29	5.83E-04	−1.04	0.32	6.05E-04	−1.11	0.28	3.96E-05
Chr9	26364751	26365250	−0.95	0.19	8.90E-07	−0.96	0.28	2.78E-04	1.13	0.30	9.90E-05	0.96	0.28	2.77E-04
Chr1	25227001	25227500	−0.83	0.17	9.05E-07	−0.87	0.25	3.81E-04	0.78	0.25	1.39E-03	0.99	0.25	5.78E-05
Chr13	68877251	68877750	0.97	0.2	9.43E-07	1.01	0.28	1.44E-04	−1.24	0.28	1.05E-05	−0.84	0.28	1.39E-03
Chr9	89126501	89127000	−0.92	0.19	9.59E-07	−1.14	0.26	9.49E-06	1.10	0.28	6.62E-05	0.76	0.26	2.12E-03
Chr13	35317501	35318000	−0.96	0.2	9.72E-07	−0.54	0.29	4.44E-02	0.54	0.31	6.52E-02	1.04	0.27	7.61E-05
Chr21	19575001	19575500	0.96	0.19	9.92E-07	0.88	0.27	8.63E-04	−0.96	0.30	8.25E-04	−1.13	0.26	1.24E-05
Chr2	169470001	169470500	−1.02	0.21	1.06E-06	−1.31	0.28	2.08E-06	1.33	0.29	6.99E-06	1.04	0.27	5.91E-05
Chr12	4310251	4310750	−0.99	0.2	1.06E-06	−0.91	0.29	8.79E-04	1.02	0.30	4.54E-04	0.87	0.30	1.99E-03
Chr6	157136501	157137000	−0.96	0.19	1.15E-06	−0.78	0.27	2.19E-03	0.91	0.30	1.37E-03	1.30	0.25	1.62E-07
Chr14	104067251	104067750	0.71	0.14	1.17E-06	0.81	0.21	7.35E-05	−0.75	0.22	3.89E-04	−0.58	0.24	9.44E-03

### Validation of IVF-DMRs

We pursued validation of the differential methylation signals at the top associated DMR (located ~3 kb upstream of *TNP1*) and at the third-ranked DMR (located in *C9orf3*), both in or near genes previously linked to infertility. Altogether, four CpG sites were targeted for validation using Sequenom’s EpiTYPER technology.

For the DMR in *C9orf3*, we were able to target two CpG sites within the most-associated 500-bp bin in this locus (Additional file 1: Figure S3). We assayed methylation levels in 36 MZ twins included in the discovery EWAS and observed significantly higher methylation in the IVF group, concordant with the MeDIP-seq analysis, at both tested CpG sites in the *C9orf3 locus* (*P* = 0.02 and 0.03, respectively), therefore validating this signal using a different methylation profiling approach (Additional file 1: Figure S4).

For the *TNP1* DMR we were unable to target CpGs within the most associated 500-bp bin, and we therefore selected two of the closest CpG sites contained within the second most associated DMR in that locus (Additional file 1: Figure S3). Within the sample of 36 MZ twins we also observed higher methylation in the IVF group, consistent with the MeDIP-seq signal, with effects close to nominal significance (*P* = 0.08; Additional file 1: Figure S4). However, correlation between the MeDIP-seq signal at the most-associated DMR in *TNP1* and the EpiTYPER methylation values was, as expected, relatively low as we were unable to target CpG sites within this most-associated DMR (correlation of 0.18 and 0 at the two tested CpG sites). We profiled additional samples from DZ twin pairs but did not obtain validation of the signal.

We also considered the effect of ICSI compared to conventional IVF in MZ twins in the validation dataset. We observed significantly higher methylation in the ICSI group at the first CpG of the targeted region near *TNP1* and at the first CpG site of *C9orf3* (Additional file 1: Figure S5).

Lastly, we also compared methylation in relation to conception method at the *H19 CTCF6* DMR in a reduced subset of CBMCs samples (n = 42 twins) using EpiTYPER. When comparing IVF to non-IVF twins (Additional file 1: Figure S6) we observed a difference with the same direction of effect as in the MeDIP-seq analysis, although not significant (*P* = 0.19). Interestingly, when comparing naturally conceived twins to twins that were conceived with any type of medical help (Additional file 1: Figure S6), i.e. not exclusively IVF, the difference reached nominal significance (*P* = 0.04), suggesting that differential methylation at this region is associated with parental subfertility rather than IVF conception.

## Discussion

Since IVF procedures are carried out during an important period of epigenetic reprogramming in early development, we hypothesised that IVF may induce epigenetic differences that persist to birth. We were able to identify significant and suggestive DMRs related to IVF conception (IVF-DMRs) in WBCs, although our results suggest that at least some of these changes may be linked to parental subfertility, which is confounded with IVF treatment. The observation that IVF-DMRs were identified close to genes implicated in fertility and reproduction suggests that a genetic signature influencing DNA methylation could be transmitted from parent to offspring. To assess this further, we estimated the heritability of the IVF-DMRs. We observed that the IVF-DMR located in *C9orf3*, a gene associated with polycystic ovary syndrome, was estimated to have a heritability at 25% and eight other FDR 25% WBC IVF-DMRs showed heritability greater than this (Fig. 3).

Epigenetic states of metastable epialleles in mammals are mitotically inherited after establishment in early development, therefore shared across tissues, and can cause expression variability within isogenic individuals [45]. A study in humans looking for systematic inter-individual variation in DNA methylation across tissues from two different lineages identified 109 candidate metastable epialleles [40]. Nutritional conditions during conception have been shown to be important to the establishment of epigenetic states at some of these metastable epialleles [46]. If an influence of IVF on the epigenetic marks of these alleles exists, it could potentially cause long lasting effects. A previous study, which included newborns from single and multiple pregnancies, identified DNA methylation differences in IVF conception at candidate metastable epialleles, although at different epialleles to those affected by maternal nutritional factors [19]. In our study, none of the 109 candidate metastable epialleles overlapped with the 46 FDR 25% WBC IVF-DMRs. This discrepancy could be attributed to differences between single and multiple pregnancies or to low power to detect such changes.

Our results also showed that IVF-DMRs, including hypomethylation of the regulatory region of *H19*, were generally not shared between WBCs and CBMCs. This observation suggests that the epigenetic differences reported here likely did not appear during early development or that these effects are not fixed and can revert in a cell type-specific manner. CBMCs, in contrast to WBCs, lack the granulocyte fraction, which is the predominant group of cells in the blood. Thus, the IVF-DMRs may be granulocyte-specific or at least in part influenced by this group of cells.

To date, there has been mixed evidence on the effect of IVF at imprinted genes and their regulatory regions. Some studies have reported DNA methylation changes or increased variability at these imprinted regions [14–16], while others have reported no associated changes [47, 48]. We observed that there is not an overall destabilisation of methylation patterns in ICRs, but specific DMRs, such as the *H19* DMR, can show a weak but nominally significant association with the method of conception. Previous studies have reported similar observations, that is, changes in methylation at some imprinted regions, but not in the majority [19, 48]. It is unknown if these changes occur due to IVF since imprinting defects have been previously described in sperm of infertile men, including hypomethylation of the *H19 CTCF6* DMR [49]. Loke et al. [16] reported that hypomethylation at this locus in buccal epithelium of newborns in the IVF group was driven by the subgroup conceived by ICSI. However, it is difficult to dissect whether the observed effect on DNA methylation of ICSI-conceived newborns is due to the technique itself or to male infertility. Whitelaw et al. [50] found higher levels of *SNRPN* methylation in buccal cells of ICSI-conceived newborns and these were associated with longer duration of infertility in the parents. In our data, we observed that the difference at the *H19 CTCF6* DMR was greater when considering any type of medical help during conception, supporting the idea that parental subfertility is the driver of methylation changes at this region. Information about the indication for assisted reproductive technology, the use of donor eggs or sperm, and the fertility status of parents in the control group would be required to further assess the effect of parental subfertility.

Adverse perinatal outcomes and increased frequency of imprinting disorders have also been observed in offspring of couples with a history of subfertility that were able to conceive naturally [51–53]. However, studies that controlled for parental subfertility by comparing siblings in which one was conceived naturally and the other by IVF also observed an effect [54]. It is likely, therefore, that both parental subfertility and IVF may induce epigenetic changes, as observed in another genome-wide study that found DNA methylation differences between IVF-conceived newborns and a group conceived through intrauterine insemination (infertile controls), but also between the latter and naturally conceived newborns (fertile controls) [19]. In addition, a study looking at 37 candidate CpG sites identified seven that were differentially methylated when comparing an IVF-conceived group born to parents without male infertility that used donor oocytes to naturally conceived newborns [55].

Finally, two IVF-DMRs associated with infertility (*TNP1* and *C9orf3*) were targeted for validation. Differential methylation was validated at the *C9orf3* gene. However, validation of the *TNP1* region was hampered by our inability to target CpG sites within the most-associated DMR in this locus. We attempted validation at *TNP1* by targeting CpG sites in the neighbouring 500-bp bin and observed consistent direction of association close to nominal significance.

In this study the non-IVF group included a set of twins conceived after GIFT and another set conceived after ovarian stimulation not followed by IVF. GIFT and ovarian stimulation are fertility treatments not equivalent to IVF since fertilisation still occurs in the fallopian tubes. We showed that our results were not affected by the inclusion of these data, potentially because they were represented in small numbers, only four out of 58 samples.

### Limitations

There are several limitations in this study. First, it is known that cell composition may represent a confounding variable in EWAS [56]. Our results use principal component analysis anticipating that these will capture cell heterogeneity, and follow-up of our findings in a subset of twins with available cell counts showed that the majority of findings remained significant after adjustment for cell heterogeneity. Second, although MeDIP-seq has the strength of genome-wide coverage, it lacks base-pair resolution, instead generating methylation scores across genomic regions. However, it has been reported that methylation of neighbouring CpG sites is correlated over distances up to 1000 bp [57], suggesting that the approach may be able to capture a good proportion of the methylation variance in a genomic region. Third, although this study includes a sample size larger than most previous studies exploring IVF, contemporary EWAS study designs generally require larger numbers of cases and controls to achieve sufficient power to detect small to moderate effect sizes [21, 58]. Lastly, our approach cannot conclusively determine the cause of the observed IVF-associated methylation changes. Future studies of IVF-associated regions in animal models, where genetic differences and infertility diseases can be discarded, could help identify if these changes were caused by IVF itself.

## Conclusions

We observed evidence for differences in DNA methylation between IVF and non-IVF twins on a genome-wide scale. A strength of this study design is that it allowed us to also estimate the contribution of genetic and environmental factors towards DNA methylation levels at the IVF-associated loci. The inclusion of only twin pregnancies also avoided biases present in studies that consider single and multiple pregnancies together. Multiple pregnancies are more common after IVF. Therefore, the differences observed when studying singleton and twin births together may be confounded with the higher risks of adverse perinatal outcomes in multiple pregnancy births, rather than IVF itself. Nevertheless, we were unable to dissect whether methylation changes were likely caused by IVF, or were due to the underlying parental subfertility, or other factors. These scenarios require further study exploring the stability of these DMRs over time, their relation with gene expression, and their potential role in health and disease.

## Additional file

Additional file 1: Supplementary tables and figures. **Table S1** Pregnancy complications and other outcomes. **Table S2** PCR assays. **Table S3** Re-analysis of FDR 25% WBC IVF-DMRs excluding other fertility treatments from non-IVF group. **Table S4** Re-analysis of FDR 25% WBC IVF-DMRs in subset with cell counts. **Figure S1** *C9orf3* FDR 25% IVF-DMR. **Figure S2** *H19 CTCF6* IVF-DMR replication. **Figure S3** CpG sites targeted for validation. **Figure S4** IVF versus non-IVF DNA methylation differences using Sequenom’s EpiTYPER technology. **Figure S5** ICSI versus conventional IVF DNA methylation differences using Sequenom’s EpiTYPER technology. **Figure S6** *H19 CTCF6* IVF-DMR replication using Sequenom’s EpiTYPER technology. (DOCX 104 kb)

## Abbreviations

BMI: Body mass index
CBMC: Cord blood mononuclear cell
DMR: Differentially methylated region
DZ: Dizygotic
EWAS: Epigenome-wide association scans
FDR: False discovery rate
GIFT: Gamete intra-fallopian transfer
ICR: Imprinting control region
ICSI: Intracytoplasmic sperm injection
IVF: *In vitro* fertilisation
MeDIP-seq: Methylated DNA immunoprecipitation coupled with sequencing
MZ: Monozygotic
PETS: Peri/postnatal Epigenetic Twins Study
SD: Standard deviation
WBC: Whole blood cell
