# Supplementary material for: DNA methylation changes at infertility genes in newborn twins conceived by in vitro fertilisation
Source: Genome Med. 2017 Mar 24;9:28. doi: 10.1186/s13073-017-0413-5 (PMC5364659; doi:10.1186/s13073-017-0413-5)
Supplement: Additional file 1: — Supplementary tables and figures. Table S1 Pregnancy complications and other outcomes. Table S2 PCR assays. Table S3 Re-analysis of FDR 25% WBC IVF-DMRs excluding other fertility treatments from non-IVF group. Table S4 Re-analysis of FDR 25% WBC IVF-DMRs in subset with cell counts. Figure S1 C9orf3 FDR 25% IVF-DMR. Figure S2 H19 CTCF6 IVF-DMR replication. Figure S3 CpG sites targeted for validation. Figure S4 IVF versus non-IVF DNA methylation differences using Sequenom’s EpiTYPER technology. Figure S5 ICSI versus conventional IVF DNA methylation differences using Sequenom’s EpiTYPER technology. Figure S6 H19 CTCF6 IVF-DMR replication using Sequenom’s EpiTYPER technology. (DOCX 104 kb) [file 13073_2017_413_MOESM1_ESM.docx]

**Supplementary tables**

Table S1. Pregnancy complications and other outcomes

|  | **Occurrence (N)** | |
| --- | --- | --- |
| **Pregnancy complications** | **non-IVF** | **IVF** |
| Gestational diabetes | 0 | 1 |
| Vacuum-assisted vaginal delivery | 0 | 1 |
| Intrauterine growth restriction | 1 | 2 |
| Shortened cervix | 1 | 1 |
| Elevated blood pressure | 0 | 2 |
| Twin-to-twin transfusion syndrome | 1 | 0 |
| Hyperemesis | 1 | 0 |
| Down syndrome | 1 | 0 |

Table S2. PCR assays

|  | Assay 1 (*TNP1*) | Assay 2 (*C9orf3*) |
| --- | --- | --- |
| Location | chr2:217727235-217727594 | chr9:97503908-97504381 |
| Product size | 360 | 474 |
| Number of analysable CpG sites | 2 | 2 |
| Left primer | aggaagagagTTGTGTGAAATTATGTTTTATGTTTGT | aggaagagagATTTTATTTTTAGTGGTATGGTTTT |
| Right primer | cagtaatacgactcactatagggagaaggctCCCTACCTTAAAATAACCCCACTTA | cagtaatacgactcactatagggagaaggctACCTTCTAAATAAAACTCCCTATATAATC |
| PCR conditions | 95°C for 10 min  5 cycles of 95°C for 20 s, 59-60°C for 30 s, and 72°C for 2 min  40 cycles of 95°C for 20 s, 59-60°C for 30 s, and 72°C for 2 min  72°C for 10 min | 95°C for 10 min  5 cycles of 95°C for 20 s, 56-58°C for 30 s, and 72°C for 2 min  40 cycles of 95°C for 20 s, 56-58°C for 30 s, and 72°C for 2 min  72°C for 10 min |

Table S3. Re-analysis of FDR 25% WBC IVF-DMRs excluding other fertility treatments from non-IVF group (n=94, 54 non-IVF and 40 IVF)

| Chromosome | Start | End | Estimate | SE | p |
| --- | --- | --- | --- | --- | --- |
| chr2 | 217726751 | 217727250 | 1.17 | 0.19 | 9.66E-09 |
| chr5 | 178761751 | 178762250 | -1.09 | 0.20 | 5.69E-08 |
| chr9 | 97504001 | 97504500 | 1.05 | 0.20 | 1.22E-07 |
| chr5 | 9275751 | 9276250 | 1.09 | 0.19 | 1.17E-07 |
| chr4 | 184814001 | 184814500 | -0.94 | 0.18 | 2.00E-07 |
| chr5 | 142488501 | 142489000 | -1.09 | 0.20 | 1.05E-07 |
| chr9 | 118148751 | 118149250 | 1.05 | 0.20 | 3.42E-07 |
| chr9 | 118149001 | 118149500 | 1.04 | 0.20 | 1.52E-07 |
| chr11 | 82654251 | 82654750 | -1.07 | 0.20 | 1.57E-07 |
| chr19 | 6165251 | 6165750 | 0.93 | 0.18 | 2.38E-07 |
| chr1 | 85522251 | 85522750 | 0.97 | 0.18 | 3.36E-07 |
| chr17 | 42569001 | 42569500 | -1.10 | 0.19 | 7.18E-08 |
| chr4 | 141606501 | 141607000 | 1.07 | 0.20 | 5.32E-07 |
| chr5 | 137736001 | 137736500 | -1.02 | 0.20 | 4.39E-07 |
| chr5 | 150614501 | 150615000 | -1.05 | 0.20 | 3.18E-07 |
| chr17 | 36918251 | 36918750 | -1.11 | 0.21 | 4.66E-07 |
| chr6 | 126138251 | 126138750 | -1.01 | 0.19 | 1.00E-07 |
| chr7 | 144431251 | 144431750 | 1.00 | 0.19 | 6.16E-07 |
| chr12 | 70937251 | 70937750 | 0.90 | 0.18 | 5.51E-07 |
| chr4 | 141606251 | 141606750 | 1.02 | 0.19 | 3.96E-07 |
| chr13 | 90019001 | 90019500 | 1.08 | 0.20 | 1.77E-07 |
| chr11 | 74179001 | 74179500 | 1.08 | 0.20 | 2.81E-07 |
| chr12 | 99153001 | 99153500 | -0.99 | 0.20 | 8.01E-07 |
| chr2 | 223336751 | 223337250 | -1.01 | 0.20 | 4.73E-07 |
| chr8 | 120972001 | 120972500 | 1.01 | 0.19 | 1.51E-07 |
| chr17 | 38047001 | 38047500 | -1.06 | 0.20 | 5.53E-07 |
| chr4 | 64626751 | 64627250 | -0.99 | 0.19 | 5.24E-07 |
| chr16 | 87256751 | 87257250 | -0.83 | 0.17 | 9.12E-07 |
| chr19 | 10656751 | 10657250 | -1.02 | 0.20 | 4.44E-07 |
| chr7 | 2487251 | 2487750 | 0.78 | 0.16 | 1.36E-06 |
| chr11 | 74178751 | 74179250 | 1.01 | 0.20 | 5.79E-07 |
| chr10 | 119176501 | 119177000 | -1.05 | 0.21 | 8.45E-07 |
| chr22 | 34755251 | 34755750 | -0.98 | 0.20 | 1.25E-06 |
| chr6 | 161664751 | 161665250 | 0.97 | 0.19 | 1.04E-06 |
| chr16 | 17161751 | 17162250 | -1.02 | 0.20 | 6.74E-07 |
| chr18 | 23695001 | 23695500 | 1.02 | 0.21 | 1.07E-06 |
| chr9 | 26364751 | 26365250 | -0.96 | 0.20 | 8.40E-07 |
| chr1 | 25227001 | 25227500 | -0.82 | 0.17 | 1.82E-06 |
| chr13 | 68877251 | 68877750 | 0.98 | 0.20 | 1.61E-06 |
| chr9 | 89126501 | 89127000 | -0.93 | 0.19 | 6.93E-07 |
| chr13 | 35317501 | 35318000 | -0.97 | 0.19 | 6.46E-07 |
| chr21 | 19575001 | 19575500 | 1.00 | 0.19 | 3.04E-07 |
| chr2 | 169470001 | 169470500 | -1.03 | 0.20 | 6.63E-07 |
| chr12 | 4310251 | 4310750 | -0.96 | 0.20 | 2.47E-06 |
| chr6 | 157136501 | 157137000 | -0.97 | 0.19 | 1.27E-06 |
| chr14 | 104067251 | 104067750 | 0.70 | 0.14 | 1.78E-06 |

Table S4. Re-analysis of FDR 25% WBC IVF-DMRs in subset with cell counts

|  |  |  | Adjusted for 5 PCs (n=98, 40 IVF and 58 non-IVF) | | | Adjusted for 5 PCs (n=54, 22 IVF and 32 non-IVF) | | | Adjusted for cell proportions (n=54, 22 IVF and 32 non-IVF) | | |
| --- | --- | --- | --- | --- | --- | --- | --- | --- | --- | --- | --- |
| Chromosome | Start | End | Estimate | SE | p | Estimate | SE | p | Estimate | SE | p |
| chr2 | 217726751 | 217727250 | 1.18 | 0.19 | 2.30E-09 | 0.98 | 0.28 | 2.78E-04 | 0.91 | 0.27 | 4.43E-04 |
| chr5 | 178761751 | 178762250 | -1.08 | 0.2 | 5.43E-08 | -1.19 | 0.26 | 3.37E-06 | -1.1 | 0.26 | 1.44E-05 |
| chr9 | 97504001 | 97504500 | 1.07 | 0.2 | 5.83E-08 | 1.14 | 0.28 | 1.85E-05 | 0.91 | 0.27 | 4.56E-04 |
| chr5 | 9275751 | 9276250 | 1.09 | 0.19 | 5.86E-08 | 1.17 | 0.25 | 5.74E-06 | 1.11 | 0.25 | 6.28E-06 |
| chr4 | 184814001 | 184814500 | -0.96 | 0.18 | 7.95E-08 | -1.06 | 0.22 | 1.00E+00 | -1.06 | 0.26 | 6.25E-05 |
| chr5 | 142488501 | 142489000 | -1.11 | 0.2 | 8.73E-08 | -1.04 | 0.29 | 2.14E-04 | -0.93 | 0.3 | 1.02E-03 |
| chr9 | 118148751 | 118149250 | 1.09 | 0.2 | 9.20E-08 | 1 | 0.29 | 3.18E-04 | 0.91 | 0.3 | 1.18E-03 |
| chr9 | 118149001 | 118149500 | 1.08 | 0.2 | 1.04E-07 | 1.02 | 0.3 | 2.41E-04 | 0.94 | 0.3 | 8.19E-04 |
| chr11 | 82654251 | 82654750 | -1.05 | 0.19 | 1.30E-07 | -1.1 | 0.26 | 1.20E-05 | -1.08 | 0.26 | 2.99E-05 |
| chr19 | 6165251 | 6165750 | 0.94 | 0.18 | 1.40E-07 | 1.05 | 0.24 | 7.97E-06 | 0.82 | 0.28 | 2.11E-03 |
| chr1 | 85522251 | 85522750 | 0.99 | 0.18 | 1.43E-07 | 0.85 | 0.28 | 1.07E-03 | 0.97 | 0.26 | 1.24E-04 |
| chr17 | 42569001 | 42569500 | -1.07 | 0.19 | 1.64E-07 | -0.97 | 0.28 | 2.15E-04 | -0.72 | 0.29 | 7.61E-03 |
| chr4 | 141606501 | 141607000 | 1.09 | 0.19 | 2.03E-07 | 0.81 | 0.26 | 1.22E-03 | 0.54 | 0.28 | 3.38E-02 |
| chr5 | 137736001 | 137736500 | -1.03 | 0.19 | 2.06E-07 | -1.06 | 0.27 | 7.31E-05 | -0.79 | 0.29 | 2.85E-03 |
| chr5 | 150614501 | 150615000 | -1.08 | 0.21 | 2.14E-07 | -1.14 | 0.28 | 2.43E-05 | -1.3 | 0.26 | 1.86E-06 |
| chr17 | 36918251 | 36918750 | -1.1 | 0.21 | 2.32E-07 | -1.12 | 0.31 | 2.47E-04 | -0.75 | 0.27 | 3.45E-03 |
| chr6 | 126138251 | 126138750 | -0.99 | 0.19 | 2.36E-07 | -1.23 | 0.25 | 7.72E-07 | -1.19 | 0.24 | 1.05E-06 |
| chr7 | 144431251 | 144431750 | 1.02 | 0.19 | 2.70E-07 | 1.12 | 0.27 | 4.08E-05 | 1.21 | 0.27 | 7.42E-06 |
| chr12 | 70937251 | 70937750 | 0.88 | 0.17 | 2.76E-07 | 0.87 | 0.25 | 4.24E-04 | 0.78 | 0.28 | 2.30E-03 |
| chr4 | 141606251 | 141606750 | 1.01 | 0.19 | 2.80E-07 | 0.76 | 0.28 | 3.19E-03 | 0.56 | 0.28 | 2.97E-02 |
| chr13 | 90019001 | 90019500 | 1.06 | 0.2 | 2.84E-07 | 1 | 0.28 | 1.44E-04 | 1.02 | 0.26 | 6.44E-05 |
| chr11 | 74179001 | 74179500 | 1.07 | 0.2 | 2.92E-07 | 0.9 | 0.28 | 4.89E-04 | 0.86 | 0.28 | 1.27E-03 |
| chr12 | 99153001 | 99153500 | -1.01 | 0.2 | 3.93E-07 | -0.97 | 0.24 | 4.62E-05 | -0.96 | 0.27 | 2.30E-04 |
| chr2 | 223336751 | 223337250 | -1.01 | 0.2 | 4.47E-07 | -0.99 | 0.27 | 8.56E-05 | -0.78 | 0.28 | 3.05E-03 |
| chr8 | 120972001 | 120972500 | 0.97 | 0.19 | 4.98E-07 | 0.53 | 0.23 | 1.21E-02 | 0.4 | 0.28 | 9.42E-02 |
| chr17 | 38047001 | 38047500 | -1.04 | 0.2 | 5.25E-07 | -1.34 | 0.26 | 2.51E-06 | -1.34 | 0.25 | 3.52E-06 |
| chr4 | 64626751 | 64627250 | -0.99 | 0.19 | 5.45E-07 | -0.95 | 0.25 | 1.07E-04 | -0.74 | 0.32 | 1.19E-02 |
| chr16 | 87256751 | 87257250 | -0.83 | 0.16 | 5.51E-07 | -1.06 | 0.19 | 7.18E-08 | -1.06 | 0.28 | 7.47E-05 |
| chr19 | 10656751 | 10657250 | -1.03 | 0.2 | 5.77E-07 | -1.06 | 0.27 | 4.01E-05 | -0.86 | 0.26 | 4.40E-04 |
| chr7 | 2487251 | 2487750 | 0.8 | 0.16 | 5.85E-07 | 0.82 | 0.19 | 1.74E-05 | 0.29 | 0.29 | 2.64E-01 |
| chr11 | 74178751 | 74179250 | 0.99 | 0.2 | 6.82E-07 | 0.89 | 0.28 | 8.08E-04 | 0.88 | 0.28 | 9.17E-04 |
| chr10 | 119176501 | 119177000 | -1.04 | 0.2 | 6.87E-07 | -1.18 | 0.27 | 1.49E-05 | -1.03 | 0.27 | 1.09E-04 |
| chr22 | 34755251 | 34755750 | -0.98 | 0.2 | 7.32E-07 | -0.85 | 0.29 | 1.70E-03 | -0.81 | 0.26 | 1.09E-03 |
| chr6 | 161664751 | 161665250 | 0.98 | 0.19 | 7.42E-07 | 0.94 | 0.3 | 1.05E-03 | 0.62 | 0.31 | 2.70E-02 |
| chr16 | 17161751 | 17162250 | -1.01 | 0.2 | 7.80E-07 | -0.94 | 0.25 | 1.13E-04 | -0.87 | 0.28 | 1.52E-03 |
| chr18 | 23695001 | 23695500 | 1.04 | 0.21 | 8.39E-07 | 0.79 | 0.33 | 1.18E-02 | 0.4 | 0.29 | 1.20E-01 |
| chr9 | 26364751 | 26365250 | -0.95 | 0.19 | 8.90E-07 | -1.02 | 0.26 | 4.45E-05 | -0.78 | 0.26 | 1.28E-03 |
| chr1 | 25227001 | 25227500 | -0.83 | 0.17 | 9.05E-07 | -0.71 | 0.22 | 4.89E-04 | -1.04 | 0.28 | 2.73E-04 |
| chr13 | 68877251 | 68877750 | 0.97 | 0.2 | 9.43E-07 | 1.07 | 0.25 | 1.21E-05 | 0.9 | 0.29 | 1.08E-03 |
| chr9 | 89126501 | 89127000 | -0.92 | 0.19 | 9.59E-07 | -0.57 | 0.25 | 1.36E-02 | -0.64 | 0.3 | 2.33E-02 |
| chr13 | 35317501 | 35318000 | -0.96 | 0.2 | 9.72E-07 | -0.77 | 0.29 | 3.49E-03 | -0.57 | 0.29 | 3.03E-02 |
| chr21 | 19575001 | 19575500 | 0.96 | 0.19 | 9.92E-07 | 1.13 | 0.22 | 3.90E-07 | 0.76 | 0.24 | 7.41E-04 |
| chr2 | 169470001 | 169470500 | -1.02 | 0.21 | 1.06E-06 | -1.12 | 0.29 | 1.22E-04 | -0.88 | 0.28 | 1.96E-03 |
| chr12 | 4310251 | 4310750 | -0.99 | 0.2 | 1.06E-06 | -0.99 | 0.29 | 2.65E-04 | -0.8 | 0.29 | 1.00E+00 |
| chr6 | 157136501 | 157137000 | -0.96 | 0.19 | 1.15E-06 | -0.98 | 0.24 | 1.88E-05 | -1.01 | 0.26 | 4.46E-05 |
| chr14 | 104067251 | 104067750 | 0.71 | 0.14 | 1.17E-06 | 0.73 | 0.2 | 1.42E-04 | 0.19 | 0.3 | 4.84E-01 |

**Supplementary figures**

Figure S1. *C9orf3* FDR 25% IVF-DMR. Boxplot of the methylation values (RPM) at the *C9orf3* FDR 25 % IVF-DMR in WBCs.

Figure S2. *H19 CTCF6* IVF-DMR replication. Boxplot of the methylation values (RPM) at the sixth CTCF binding site within the *H19* DMR (*H19 CTCF6*).

Figure S3. CpG sites targeted for validation. Top panel shows EWAS signals neighbouring the most associated DMR located near *TNP1* (a) and *C9orf3* (b) genes. Bottom panel shows the corresponding 500bp bins tested during EWAS, the CpG sites contained in those regions, and the targeted region for PCR.

Figure S4. IVF vs non-IVF DNA methylation differences using Sequenom’s EpiTYPER technology. Horizontal lines show the group median.

Figure S5. ICSI vs conventional IVF DNA methylation differences using Sequenom’s EpiTYPER technology. Horizontal lines show the group median.

Figure S6. *H19 CTCF6* IVF-DMR replication using Sequenom’s EpiTYPER technology. Boxplot of the methylation values at the sixth CTCF binding site within the *H19* DMR (*H19 CTCF6*) comparing IVF vs non-IVF newborns (left) and the group using any type of medical help vs naturally conceived newborns (right).
